# Utility of molecular and serodiagnostic tools in cerebral toxoplasmosis with and without tuberculous meningitis in AIDS patients: A study from South India

**DOI:** 10.4103/0972-2327.74197

**Published:** 2010

**Authors:** Sreenivas Adurthi, Anita Mahadevan, Radhika Bantwal, Parthasarthy Satishchandra, Sujay Ramprasad, Hema Sridhar, S. K. Shankar, Avindra Nath, R. S. Jayshree

**Affiliations:** Department of Microbiology, Kidwai Memorial Institute of Oncology, Bangalore, India; 1 Department of Neuropathology, National Institute of Mental Health and Neurosciences, Bangalore, India; 2 Department of Neurology, National Institute of Mental Health and Neurosciences, Bangalore, India; 3 Anand Institute of Laboratory Medicine, Bangalore, India; 4 Department of Neurology, Johns Hopkins University, Baltimore, Maryland, USA

**Keywords:** B1 gene, cerebral toxoplasmosis, human immunodeficiency virus, *T. gondii* IgG, *Toxoplasma gondii*, tuberculous meningitis

## Abstract

**Background:**

Antemortem diagnosis of cerebral toxoplasmosis, the second most common opportunistic infection (OI) in HIV-infected individuals in developing countries is a challenge.

**Materials and Methods:**

*Toxoplasma gondii (T.gondii)* -specific serology and nested polymerase chain reaction (nPCR) were evaluated in sera and ventricular/lumbar cerebrospinal fluid (CSF) of 22 autopsy confirmed cases of cerebral toxoplasmosis with HIV and 17 controls. Frequency of concomitant *T.gondii* infection was investigated in 17 cases of HIV-associated tuberculous meningitis (TBM).

**Results:**

The sensitivity, specificity, and positive and negative predictive values of *T. gondii* IgG on CSF (ventricular and lumbar) and sera was 100% in histology proven cerebral toxoplasmosis (concentrations: 258 ± 50, 231 ± 36, and 646 ± 243 IU/mL, respectively); majority (94%) being high avidity type, suggesting reactivation/reinfection. The sensitivity of B1 nPCR was 100% on ventricular CSF, whereas it was only 77% on lumbar CSF. Based on histology, nPCR, and IgG serology, *T. gondii* co-infection with TBM was observed in 65% (11/17) of cases.

**Discussion and Conclusion:**

CSF IgG serology and nPCR are tests with high sensitivity and specificity for the diagnosis of cerebral toxoplasmosis. TBM and cerebral toxoplasmosis can coexist and should be considered in the background of HIV infection in developing countries.

## Introduction

Cerebral toxoplasmosis is the most common cause of focal brain lesions in patients with Acquired Immuno Deficiency Syndrome (AIDS).[1] In the USA and Western Europe, 10%–30% of seropositive AIDS patients die of cerebral toxoplasmosis.[2] Availability of Highly Active Anti-Retroviral Therapy (HAART) has decreased the incidence of opportunistic infections (OIs) of the central nervous system (CNS) in AIDS patients in the West. However, these infections still remain important causes of morbidity and mortality in the developing countries.[3,4] A definitive diagnosis of cerebral toxoplasmosis relies on histologic demonstration of tachyzoites in the biopsies of brain lesions. Although a presumptive diagnosis is based on clinical signs and symptoms, demonstration of antibodies to *Toxoplasma gondii (T.gondii)* in the cerebrospinal fluid (CSF) and/or serum in the presence of mass lesions with perilesional edema on cranial CT scan/MRI is considered diagnostic. Neuroimaging facilities are not widely available in all centers or are unaffordable. Hence there is a requirement for alternative tools for the diagnosis of cerebral toxoplasmosis.[3] We undertook a retrospective study on autopsy confirmed cases of HIV-associated cerebral toxoplasmosis to evaluate toxoplasma-specific serology and B1 gene nested polymerase chain reaction (nPCR) on sera and CSF. A second aim was to assess the frequency of concomitant cerebral toxoplasmosis in a cohort of bacteriologically and autopsy confirmed cases of tuberculous meningitis (TBM), which is endemic in South India. This co-infection may modulate the clinical progression of HIV and its biological behavior.

## Materials and Methods

### Patients

Sera and CSF samples analyzed in the study were collected at autopsy with informed consent of close relatives of the deceased. At the time of autopsy, sterile lumbar puncture needles and disposable syringes were used for sample collection to prevent cross contamination. As a practice, following clinical autopsies from cases suspected of HIV/AIDS with OIs, the autopsy table, instruments are routinely decontaminated. These autopsies were conducted at a frequency of one per week on an average obviating the possibility of cross contamination. Samples were aliquoted under sterile conditions in a biohazard laminar flow hood and stored at –86°C in Human Brain Tissue Repository for neurobiological studies (Human Brain Bank), in the Department of Neuropathology, National Institute of Mental Health and Neuro Sciences, Bangalore, India. The study was approved by the Institutional ethics committee. The postmortem delay from the time of death to collection and freezing of the samples varied from 3 to 20 h. The study material included CSF (lumbar spinal and cerebral ventricular) and sera (from heart blood) collected at autopsy. The cases comprised three groups. The clinical and epidemiologic details of all three groups are provided in Table 1

**Table 1:** Clinical and epidemiologic features (Group I)

ID No.	Age/sex	Clinical history	Final diagnosis
00/TB/C130	42/M	Headache, unsteady gait, weight loss, and appetite loss – 2 weeks. CT scan – diffuse cerebral edema	Toxoplasma encephalitis
98/HIV/C38	28/M	Fever with rigors – 1 week, seizures and altered sensorium – 6 days. CT scan revealed obstructive hydrocephalus	Toxoplasma encephalitis
01/HIV/C72/S50	38/M	Fever – 3 days, seizures – 2 days, slurred speech and unsteadiness – 2 days MRI – multiple hyperintense lesions in bilateral frontal, cerebellum, and brain stem	Toxoplasma encephalitis
01/HIV/C82	45/F	Cough with expectoration – 2 months. Developed headache, walking difficulty, and right hemiparesis – 1 week MRI - multiple enhancing lesions in basal ganglia, brain stem, and cerebellum	Toxoplasma encephalitis
02/HIV/C103/S77	25/M	Fever – 2 weeks. Headache, altered sensorium – 2 days with right hemiparesis. CT scan – hypodense lesions in left basal ganglia, cerebellum	Toxoplasma encephalitis
99/HIV/C42	32/M	Right hemiparesis – 8 days with slurring of speech. CT scan – multiple ring-enhancing lesions	Toxoplasma encephalitis
01/HIV/C83	30/M	Fever with cough – 1 month. Left hemiplegia – 10 days. CT scan – multiple hypodense lesions in right frontal, basal ganglia	Toxoplasma encephalitis
02/TB/C140	26/M	Headache, vomiting, fever – 1 week, altered sensorium – 1 day with right hemiparesis. CT scan – hydrocephalus. Treated for pulmonary tuberculosis – 1 year	Toxoplasma encephalitis
01/M/C298	32/F	Headache, vomiting, fever – 1 month, altered sensorium, with ataxia and swallowing difficulty –10 days. CT scan – multiple hypodensities, bilateral parieto-occipital and basal ganglia	Toxoplasma encephalitis
02/HIV/C94	42/M	Headache – 3 months with memory difficulties. Altered sensorium – 8 days. Herpes zoster over the trunk – 1 week earlier. MRI – multiple lesions in left fronto-temporal	Toxoplasma encephalitis
99/HIV/C46	23/M	Fever with cough – 20 days. Headache and vomiting – 3 months. CT scan – right frontal parietal lesion	Toxoplasma encephalitis
02/HIV/C101	40/M	Fever, headache – 3 months. Left hemiparesis with chorea – 15 days. CT scan – normal	Toxoplasma encephalitis
01/TB/C136	7/M	Fever, seizures – 15 days. Altered sensorium – 10 days. CT scan – multiple ring-enhancing lesions in bilateral frontal thalamus and brainstem	Toxoplasma encephalitis
01/HIV/C76	32/M	Left hemiparesis – 4 months, ptosis with unsteady gait – 2 months. CT scan –enhancing lesions in brainstem	Toxoplasma encephalitis
99/TB/C114	20/F	Fever, headache, left hemiparesis – 15 days. CT – multiple lesions in right basal ganglia and thalamus	Toxoplasma encephalitis
95/M/C60	20/M	Fever, headache, cough – 3 months, weight loss and appetite loss – 3 months. Seizures – 3 months. CT scan – multiple lesions in right frontal and cerebellum	Toxoplasma encephalitis
97/V/C27	32/M	Generalized seizures and altered sensorium –- 2 days. Deeply comatose on admission	Toxoplasma encephalitis
02/HIV/C91	30/F	Headache – 1 month, seizures followed by altered sensorium –1 week. Left eye ptosis CT – hypodense lesion in right basal ganglia and parietal region	Toxoplasma encephalitis
98/HIV/C40	40/M	Fever with altered sensorium – 10 days. Neck stiffness with hemiparesis. MRI –multiple lesion, left basal ganglia, cerebellum, and bilateral frontal	Toxoplasma encephalitis
00/HIV/C59	20/F	Fever, cough, weight loss –1 month. Right hemiparesis, aphasia – 8 days. CT – multiple ring-enhancing lesion, left parietal and right basal ganglia	Toxoplasma encephalitis
99/M/C171	33/F	Fever – 1½ months, headache, vomiting – 1 month followed by altered sensorium – 1 day. CT – left bilateral parietal hypodense lesion	Toxoplasma encephalitis
02/HIV/C95	30/M	Fever, headache, visual blurring – 7 days. Left focal seizures – 6 days. MRI – multiple ring-enhancing lesion, bilateral basal ganglia, frontal, and cerebellum.	Toxoplasma encephalitis

#### Group I

Twenty-two HIV-1 seropositive patients (confirmed to be clade C by subtype-specific PCR) who succumbed to cerebral toxoplasmosis manifesting as encephalitis or mass lesions were included. All the cases had histopathologically confirmed toxoplasma lesions in various stages of evolution. HIV-seronegative cerebral toxoplasmosis cases were not available in our center.

#### Group II

Seventeen HIV-seropositive cases of TBM confirmed by CSF seropositivity for IgG antibody and immune complexes to lipoarabinomannan antigen of *Mycobacterium tuberculosis (M.tuberculosis)* and/or demonstration of acid fast organisms in the smear from the basal exudates in the brain at autopsy with histopathologic evidence of chronic granulomatous meningitis (with or without caseous necrosis) were studied.

#### Group III

Seventeen HIV-seronegative age- and sex-matched cases who succumbed to road traffic accidents (without evidence of cranial trauma, identifiable neurologic disorder, or infective conditions) and whose sera were negative for *T. gondii* antibodies were used as controls. Serum and CSF samples from HIV-positive cases without OI were not available in our cohort and hence could not be included for study.

The investigators carrying out the enzyme-linked immunosorbent assay (ELISA) and nPCR studies were totally blinded to the origin of samples and the diagnosis of cases. Coded samples were provided to them, which were decoded only after completion of the study for analyses of results.

### Methodology

#### Serology

Lumbar and ventricular CSF samples and sera were tested in duplicates for IgG and IgM antibodies to *T. gondii* using commercial ELISA kits (Euroimmun, Germany) and expressed in International Units (IU)/mL. Sera were tested at a dilution of 1:100 (as recommended in the kit protocol) and CSF dilutions were fixed at 1:10 by titration. Antibody levels greater than 10 IU/mL were considered as positive. Samples positive for IgG antibodies were further tested for their avidity and the results were interpreted as high or low avidity, following manufacturer’s instructions (Euroimmun, Germany). The mean concentrations of *T. gondii*-specific IgG in CSF and sera were compared by the Student’s *t* test.

#### DNA extraction

DNA was extracted from aliquots of supernatants of 200 µL CSFs stored at –86°C, using commercial extraction kits (Qiamp Tissue Kit, Qiagen, Germany).

#### Polymerase chain reaction

Nested PCR was carried out using primers against the B1 gene of *T. gondii*.[5] The sensitivity of nPCR was determined using dilutions of *T. gondii* culture DNA (gift from Dr. Philippe Thulliez, Institut de Puériculture, Paris, and Professor M. L. Dubey, Department of Parasitology, Post Graduate Institute of Medical Education and Research, Chandigarh, India). β-Globin PCR was used for house keeping to exclude the presence of polymerase inhibitors and confirm the efficiency of DNA extraction.[6] The limit of detection of *T. gondii* DNA by nPCR was 100 fg (data not shown). PCR was negative when amplified using DNA from cultures of *Aspergillus fumigatus, M. tuberculosis*, and *Candida albicans*.

#### Performance characteristics

The performance characteristics, namely, sensitivity, specificity, and positive and negative predictive values of both nPCR and *T. gondii*-specific serology were determined on CSF and sera from groups I and III considering them as true positives and negatives, respectively.

#### Histologic study of brains for toxoplasma lesions

The brains collected at autopsy were fixed in 10% buffered formalin for 4 weeks before sectioning. Lesions identified on gross examination were sampled for paraffin embedding and histologic evaluation. The lesions were mostly located supratentorially and a few in the cerebellum and brain stem, both deep in the parenchyma and close to subarachnoid space. Brain tissue localization of *T. gondii* cysts and tachyzoites was confirmed by immunohistochemistry using commercial antibody to P30 antigen (Novocastra Laboratories Ltd., UK).

## Results

### Group I (cerebral toxoplasmosis, n=22)

**Figure 1:**
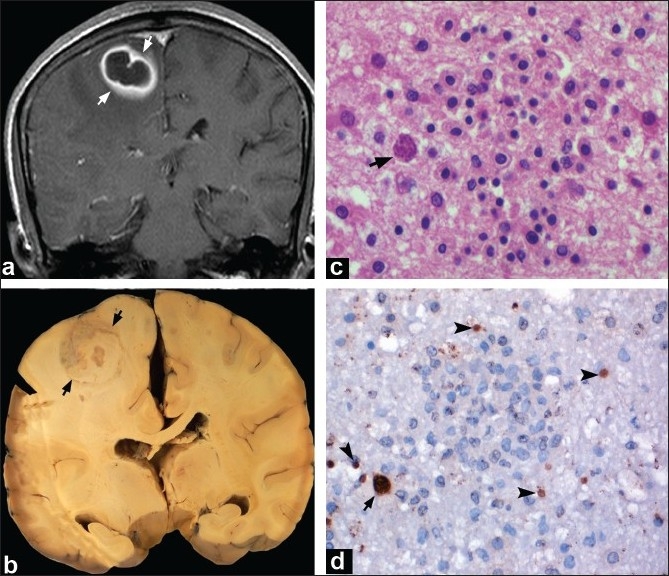
Case 10: MRI of a 50-year-old immunocompromised male revealed a ring-enhancing lesion in right parietal region (a). He received antitoxoplasma therapy for 2 days. Autopsy revealed a large organizing abscess in the right parietal region (b). Histology showed tissue cysts (arrow, c) adjacent to microglial nodule (c). Immunostaining for P30 antigen confirmed the presence of multiple tachyzoites (arrow heads) and tissue cysts (arrow) (d) of *T.gondii* Nested PCR (CSF) and IgG (CSF and serum) were positive for *T.gondii* (c: HE ×320, d: immunoperoxidase for P30 antigen ×320)

The mean age of the patients was 31 ± 9 years, (M:F=16:6). Nineteen of the 22 cases clinically manifested with signs of raised intracranial tension and focal neurologic deficits. Neuroimaging studies revealed single or multiple ring-enhancing lesions involving basal ganglia, thalamus, brain stem, and cerebral cortex. Three cases presented with features of encephalitis with headache and seizures. Neuroimaging revealed multiple necrotizing hemorrhagic lesions with diffuse cerebral edema causing mass effect and midline shift [Table 1]. At autopsy, mass lesions were found located in the frontal/parietal cortices [Figure 1a, b], basal ganglia, and brainstem with single/multiple abscesses at various stages of organization. Histologically, characteristic bradyzoites and ruptured tachyzoite forms of *T. gondii* were detectable [Figure 1c], which were confirmed immunohistochemically [Figure 1d].

In 14/22 patients with histopathologically proven cerebral toxoplasmosis, all 3 samples (lumbar CSF, ventricular CSF, and sera) were available. The mean CSF glucose was 53 ± 7 mg/dL; protein: 184 ± 63 mg/dL, and cell count of the lumbar CSF was 21 ± 7/µL.

### Group III (n=17)

Clinical and epidemiologic details are provided in Table 1. The mean age of the control group subjects was 34 ± 12 (M:F=13:4). All the samples from the control group were negative for both *M. tuberculosis*- and *T. gondii*-specific antibodies and protozoal DNA by nPCR.

### Performance characteristics

*Serology* : The sensitivity, specificity, and positive and negative predictive values were calculated from the data obtained from groups I and III. For *T. gondii*-specific IgG on all the three samples tested, these were 100% each; whereas for IgM tested on sera, these were 61%, 100%, and 100% and 71%, respectively.

In Group I [Table 2], *T. gondii*-specific IgG was present in all CSF (lumbar: 16/16, ventricular: 14/14) and sera (18/18) tested. The concentrations of IgG in the ventricular and lumbar CSF and sera ranged from 186 to 360 IU/mL (mean 258 ± 50 IU/mL), 182 to 282 IU/mL (mean 231 ± 36 IU/mL), and 196 to 1016 IU/mL (mean 646 243 IU/mL), respectively. While the concentrations in sera were significantly higher (*P* < 0.05), there was no difference in the mean concentrations of IgG in the ventricular and lumbar CSF samples (*P* > 0.05). All the patients except one (case 1) had high-avidity IgG antibodies both in the CSF and sera. IgM antibody to *T. gondii* were less frequent in CSF (1/16 in lumbar, 2/16 in ventricular) compared to serum (11/18, 61%).

**Table 2:** *T.gondii* nPCR, and specific serology—IgM, IgG, and IgG avidity in lumbar and ventricular CSFs and sera from histopathologically confirmed cases of cerebral toxoplasmosis (Group I)

ID No.	DOI (days)	nPCR		IgM			IgG			IgG avidity		
		L	V	L	V	S	L	V	S	L	V	S
00/TB/C130	15	+	+	–	–	+	194	224	792	LA	LA	LA
98/HIV/C38	6	+	+	–	–	ND	216	ND	ND	HA	ND	ND
01/HIV/C72/S50	3	+	+	–	–	–	250	260	242	HA	HA	HA
01/HIV/C82	14	+	+	–	–	+	182	220	872	HA	HA	HA
02/HIV/C103/S77	2	–	+	–	–	+	242	186	260	HA	HA	HA
99/HIV/C42	8	+	+	–	–	+	262	288	776	HA	HA	HA
01/HIV/C83	10	–	+	–	–	–	212	310	840	HA	HA	HA
02/TB/C140	7	+	+	+	+	+	186	230	832	HA	HA	HA
01/M/C298	30	+	+	–	–	+	254	360	736	HA	HA	HA
02/HIV/C94	90	+	+	–	–	–	224	270	456	HA	HA	HA
99/HIV/C46	60	–	+	–	ND	+	260	ND	700	HA	ND	HA
02/HIV/C101	90	+	+	–	–	ND	276	200	ND	HA	HA	ND
01/TB/C136	1yr/15d	+	ND	–	–	ND	282	242	ND	HA	HA	ND
01/HIV/C76	120	–	+	–	–	–	184	296	568	HA	HA	HA
99/TB/C114		+	+	–	ND	+	200	ND	856	HA	ND	HA
95/M/C60	90	ND	+	ND	–	–	ND	334	600	ND	HA	HA
97/V/C27	3	ND	+	ND	ND	+	ND	ND	1016	ND	ND	HA
02/HIV/C91	14	ND	+	ND	+	+	ND	224	392	ND	HA	HA
98/HIV/C40	10	ND	ND	ND	–	+	ND	ND	196	ND	ND	HA
00/HIV/C59		ND	ND	–	ND	ND	264	ND	ND	HA	ND	ND
99/M/C171	30	+	ND	ND	ND	–	ND	ND	704	ND	ND	HA
02/HIV/C95	7	+	ND	ND	ND	–	ND	ND	792	ND	ND	HA
Mean							231a	258b	646c			
SD							+36	+50	+243			
No. +		13/17	17/17	1/16	2/16	11/18	16/16	14/14	18/18	1LA	1LA	1LA
% +		76.50	100	6.20	12.50	61	100	100	100	15HA	13HA	17HA

DOI, duration of illness; L, lumbar; V, ventricular; CSF, cerebrospinal fluid; S, serum; ND, not done; +, positive; –, negative; HA: high avidity; LA: low avidity a vs b *P* >0.05; b vs c *P* <0.05; a vs c *P* < 0.05.

#### Polymerase chain reaction

The sensitivity, specificity, positive and negative predictive values of B1 nPCR on ventricular CSF were 100% each. However, in lumbar CSF, the sensitivity and the negative predictive values were lower at 76.5% and 81% respectively. In four cases where *T. gondii* nPCR was false negative in lumbar CSF, the pathological lesions in the brain were relatively avascular, organizing abscesses with extensive necrosis, edema and focal hemorrhage located in deep parenchyma, away from the subarachnoid space.

### Concomitant toxoplasmosis in TBM (Group II, n=17)

Mean age of the patients (n=17), in this group was 34 ± 6 years, (M:F=16:1). The patients presented with features of chronic meningitis with headache, vomiting, and neck stiffness. Neuroimaging highlighted enhancing exudates in basal cisterns partially entrapping the vessels with occasional ring-enhancing lesions in basal ganglia [Table 1]. The mean CSF glucose was 28 ± 16 mg/dL; protein: 227 136 mg/dL, and cell count of the lumbar CSF was 198 ± 275/µL [Table 3].

**Table 3:** *T.gondii* nPCR and specific serology in lumbar CSF from cases of TBM (Group II)

ID No. (DOI, days)	nPCR	IgM	IgG	IgG avidity	Pathology	Comment
00/HIV/C70/S48 (30)	–	–	+**	HA	TBM with toxoplasmosis	False-negative PCR and IgM possibly due to? intermittent shedding/cryptic lesion as no antitoxoplasma treatment received
01/HIV/C73/S51 (10)	–	–	+	HA	TBM with toxoplasmosis	False-negative PCR and IgM possibly due to? intermittent shedding/cryptic lesion as no antitoxoplasma treatment received
02/HIV/C92/S67 (3)	–	–	+	LA	TBM with toxoplasmosis	False-negative PCR and IgM due to antitoxoplasma treatment for 3 weeks
01/TB/C152/S88 (1 day)	–	–	–**	NA	TBM with toxoplasmosis	False-negative PCR and serology due to antitoxoplasma treatment for 4weeks, which could HAve abrogated local antibody response in CSF
99/HIV/C41 (6 days)	+	–	+	HA	TBM with arteritis and disseminated tuberculosis No evidence of toxoplasmosis	False-negative IgM#
99/HIV/C44 (7 days)	+	–	+**	HA	TBM with arteritis No evidence of toxoplasmosis	False-negative IgM#
98/TB/C90/S26 (30)	+	–	+**	HA	TBM with arteritis No evidence of toxoplasmosis	False-negative IgM#
98/TB/C94/S30 (10)	–	+*	+**	LA	TBM with arteritis and disseminated tuberculosis No evidence of toxoplasmosis	False-negative PCR due to primary infection, possibly extracerebral#
02/HIV/C97/S72 (30)	–	–	+**	HA	TBM with spinal arachnoiditis No toxoplasma lesions	False-negative PCR and IgM possibly due to? intermittent shedding/cryptic lesion as no antitoxoplasma treatment received#
98/HIV/C49/S31 (20)	–	–	+**	HA	TBM with disseminated TB No toxoplasma lesions	False-negative PCR and IgM possibly due to? intermittent shedding/cryptic lesion as no antitoxoplasma treatment received#
97/HIV/C22/S7 (8)	–	–	+**	HA	TBM with arteritis No toxoplasma lesions	False-negative PCR and IgM possibly due to? intermittent shedding/cryptic lesion as no antitoxoplasma treatment received#
03/TB/C151/S87 (7)	–	+*	–	NA	TBM with spinal arachnoiditis and disseminated tuberculosis No evidence of toxoplasmosis	False-positive IgM possibly due to systemic infection
95/HIV/C14 (20)	–	+*	–	NA	TBM with arteritis No evidence of toxoplasmosis	False-positive IgM possibly due to systemic infection
96/HIV/C5 (180)	–	–	–	NA	TBM with arteritis and disseminated TB No toxoplasma lesions	
00/HIV/C67 (15)	–	–	–	NA	TBM with tuberculomata and arteritis No toxoplasma lesions	
99/HIV/C51 (3)	–	–	–	NA	TBM with disseminated TB No toxoplasma lesions	
98/HIV/C39/S23 (60)	–	–	–	NA	TBM with arteritis No toxoplasma lesions	
Mean			224			
SD			78			
No. +	3/17	3/17	10/17	8 HA		
% +	18	18	59	2 LA		

TB, tuberculosis; TBM, tuberculous meningitis; HA, high avid; LA, low avid, NA, not applicable; DOI, duration of illness. – indicates negative; + indicates positive; IgG values are represented in IU/mL.

* Serum IgM also positive in these cases.

** Serum IgG positive in all.

# False-negative histology: possibly due to sampling error as toxoplasma lesions may be masked by TBM arteritic changes.

Among the patients with TBM, histologic evidence of cerebral toxoplasmosis was seen in 4 (cases 1–4). Although nPCR for *T. gondii* was negative in all 4 cases, three had parasite-specific IgG antibodies in CSF (>200 IU/mL) (cases 1–3). In one case (case 4), both CSF serology and nPCR were negative, but IgM and low-avidity IgG antibodies were present in the serum.

Of the remaining 13 cases without histologic evidence of cerebral toxoplasmosis, three showed nPCR and high titer IgG antibodies in CSF (cases 5–7), three had high IgG levels alone (cases 9–11), and one had both IgM and IgG in CSF (case 8). There were two other cases in this group in which none of the other parameters showed any evidence of cerebral toxoplasmosis except CSF IgM positivity (cases 12–13); the IgM positivity seen in this case could be labeled as false positive, since it was not accompanied by low-avidity IgG antibodies or nPCR positivity.

## Discussion

The incidence of OI of the CNS, including cerebral toxoplasmosis in the HIV-infected individuals has declined considerably with the introduction of HAART in the developed world.[7] However, in the developing countries, toxoplasmosis remains the most important cause of focal brain lesions[8] and in India, it is a close second to tuberculosis at times causing diagnostic difficulties.[9,10] This is particularly relevant considering the high seroprevalence of IgG antibodies to *T. gondii* in South Indian healthy voluntary blood donors (20.3%).[11] We evaluated the diagnostic efficacy of nPCR vis-à-vis that of conventional serodiagnostic tests in a cohort of pathologically confirmed cases of HIV-associated cerebral toxoplasmosis. We also studied the occurrence of co-infection of cerebral toxoplasmosis in pathologically confirmed cases of TBM since it has therapeutic implications.

CSF antitoxoplasma serology appears to be remarkably sensitive for the diagnosis of cerebral toxoplasmosis[3,12,13] but has relatively low specificity in discriminating between recent, active, and past dormant toxoplasma infection.[14] Intrathecal synthesis of antibodies to *T. gondii* was reported in 69% of AIDS patients with cerebral toxoplasmosis by ELISA[12] and ~50%–100% by applying advanced techniques, such as affinity-mediated immunoblot.[13] In a group of 33 clinically suspected cases of AIDS-associated cerebral toxoplasmosis from North India, 72% were reported to have high serum IgG titers.[15] In the present study from South India, in patients with pathologically confirmed infection, the sensitivity of *T. gondii* serology was 100% on both sera and CSF, suggesting antibody detection to be a useful diagnostic tool even in the HIV-induced immunosuppressive state.[16] The mean serum titers (646 ± 243 IU/mL) were significantly higher than in the CSF and much higher than the levels reported by Singh and Dubey from North India (274.8 ± 338 IU/mL).[10] Serum IgM antibodies have been reported in 2% and 9% of cases of cerebral toxoplasmosis from the USA and India, respectively.[10,14] In the present study, specific IgM antibodies were present in 61% of the sera tested (duration of illness ranged from 2 to 60 days). However, IgM may not be a reliable indicator of primary or recent *T. gondii* infection, since it is reported to persist for as long as 2 years after primary infection.[17] Reactivation of latent toxoplasma infection in the brain appears to be the norm in HIV-infected patients,[9,15,18,19] as seen in the present study.

PCR for *T. gondii* DNA in the CSF has a sensitivity varying from 0%[18] to 100%,[13,20–24] which depends on various factors.[25] We found the sensitivity of nPCR on ventricular CSF to be 100% compared with 76% on lumbar CSF, which is due to ventricular CSF being in direct contact with the toxoplasma lesions. Relatively lower sensitivity of detection of parasitic DNA in lumbar CSF could probably be attributed to limited release of the parasites[26,27] or intermittent shedding of *T. gondii* parasites into the CSF space. Hence repeated testing on sequentially collected samples is recommended.[26] Initiation of specific therapy prior to testing can significantly reduce sensitivity of PCR.[13] Of the four patients in our study with negative nPCR, only one had received specific antitoxoplasma therapy.

Co-infection of *T. gondii* with *M. tuberculosis* was detected by histology/parasite-specific serology/nPCR in 11/17 (65%) of cases. Among these, four (24%) cases had toxoplasma lesions in the brain. A single case with histologically confirmed TBM and cerebral toxoplasmosis, had IgM and low-avidity IgG antibodies present only in the serum [case 4, Table 3]. This patient had received a four-week course of antitoxoplasma therapy that could have eliminated the parasite or enclosed it in a necrotic shell producing false-negative CSF serology and nPCR. The failure to detect pathologic lesions of toxoplasmosis in three cases in which the parasite B1 gene was amplifiable by nPCR in the CSF [cases 5–7, Table 3] could be due to the toxoplasma lesions being masked by the mycobacterial meningitic process with arterial infarctions or presence of a cryptic focus that was missed in the histopathologic sampling. Local synthesis of high titers of IgG antibodies to the parasite was proof of cerebral toxoplasma infection. In conclusion, despite the incongruity, these findings suggest subclinical coexistence of toxoplasmosis in the HIV-infected in endemic areas. Identification of this co-infection is essential to institute appropriate and timely therapy in clinical management.

## Conclusion

In summary, estimation of *T. gondii*-specific IgG antibodies in sera and/or CSF appears to be a sensitive and reliable marker for the diagnosis of cerebral toxoplasmosis even in the presence of TBM. Reinfection/reactivation of dormant *T. gondii* infection appears to be the norm in association with HIV. nPCR for toxoplasma on lumbar CSF has a limited usefulness but could be a useful adjunct to serology. An important message to the treating clinicians in the developing countries is to consider the possibility of concomitant cerebral toxoplasmosis in patients with TBM, especially in the immunocompromised, as this has therapeutic implications.
